# Statistical Fragility of Randomized Controlled Trials Evaluating Platelet-Rich Plasma Use for Knee Osteoarthritis: A Systematic Review

**DOI:** 10.1177/23259671231187894

**Published:** 2023-08-21

**Authors:** Justin P Chan, Michael Vrla, Claire Thompson, David P Trofa, Xinning Li, Dean Wang, Robert L Parisien

**Affiliations:** † Department of Orthopaedic Surgery, University of California, Irvine, Irvine, California, USA.; ‡ New York University Grossman School of Medicine, New York, New York, USA.; § Department of Orthopedic Surgery, Columbia University Medical Center, New York, New York, USA.; ∥ Department of Orthopaedic Surgery, Boston University School of Medicine, Boston, Massachusetts, USA.; ¶ Department of Orthopaedic Surgery and Sports Medicine, Mount Sinai Health System, New York, New York, USA.; *Investigation performed at University of California, Irvine, Irvine, California, USA*

**Keywords:** cartilage preservation, knee osteoarthritis, orthobiologics, platelet-rich plasma

## Abstract

**Background:**

Numerous studies have been published on the use of platelet-rich plasma (PRP) for knee osteoarthritis (OA), with conflicting results.

**Purpose:**

To determine the fragility index (FI) and fragility quotient (FQ) of randomized controlled trials (RCTs) that evaluated the use of PRP to treat knee OA.

**Study Design:**

Systematic review.

**Methods:**

RCTs evaluating the efficacy of PRP injections for knee OA from 2000 to 2020 were included for analysis according to PRISMA guidelines. The FI was determined by calculating the number of outcome event reversals required to change the statistical significance. The associated FQ was determined by dividing the FI by the sample size.

**Results:**

Our initial search resulted in 41,149 studies, of which 8 RCTs (678 patients, 72 outcome events) were included in the analysis. One study failed to report PRP formulation details, whereas 87.5% of studies reported using either leukocyte-rich or leukocyte-poor PRP. The platelet concentration was reported in 25% of the included trials. The overall FI of the 72 outcome events was 8.5. Accounting for sample size, the associated FQ was determined to be 0.14, suggesting that the reversal of 14% of outcome events was required to change outcome significance. There were 51 statistically significant outcomes, of which the FI and FQ were 12 and 0.164, respectively.

**Conclusion:**

Comprehensive fragility analysis suggested that the published literature evaluating the efficacy of PRP use for knee OA may lack statistical stability. We recommend the reporting of both an FI and FQ in addition to *P* value analysis to provide a clear and thorough understanding of the statistical integrity of studies reporting on PRP use for knee OA.

Modern medicine is characterized in large part by the incorporation of evidence-based guidelines for treatment indications and techniques. As clinical research continues to advance, comprehensive evaluation of the available literature is critically important to the understanding of significant findings. The standard method of reporting statistical analyses in the literature is in the form of *P* values, with the conventional threshold set at α = .05, correlating to a 5% chance of the observed outcome occurring by chance.^3^ If the *P* value is less than .05, the null hypothesis is rejected and the observed difference is determined to be statistically significant. The *P* value is interpreted in a dichotomous fashion, representing either significance or no significance. Despite this relative ease of interpretation, it may prove more difficult to reconcile when similar studies offer conflicting significance of outcomes data. Statistical significance may also not necessarily equate to clinical significance.

The apparent reversal of significance can be determined via careful calculation of the fragility index (FI). First proposed by physician and epidemiologist Alvan Feinstein in 1990, the FI is a measure of the number of outcome event reversals necessary to alter statistical significance.^9^ The FI concept has since been applied across medical specialties with recent adoption in musculoskeletal medicine and orthopaedic surgery.^5,10,14,15,20,21^ To account for differing sample sizes, the FI is appropriately supplemented with the fragility quotient (FQ). The FQ is a measure of quantitative significance and is determined by dividing the FI by the sample size.

Knee osteoarthritis (OA) is a common source of musculoskeletal pain, with a global prevalence estimated at 3.8%.^6^ As the population ages, knee OA is predicted to become an even more frequent cause of disability, resulting in an increasing burden on individuals and a financial burden to the health care systems and societies.^6^ Total joint arthroplasty is a well-established surgical method for the treatment of end-stage OA but may only be considered in severe OA in the setting of failed nonoperative management. Physical therapy, knee unloader braces, and nonsteroidal anti-inflammatories are also mainstays of treatment. Intra-articular steroid or hyaluronic acid injections are frequently employed as well, but have demonstrated inconclusive clinical effectiveness.^11–13^

In the absence of consistently effective nonoperative treatments for knee OA, there has been a recent increase in scientific interest with regard to the development of new biologic modalities. In particular, platelet-rich plasma (PRP) is an autologous blood product containing high concentrations of growth factors, which have been hypothesized to enhance cartilage healing and reduce inflammation.^1^ There have been an increasing number of studies in the last decade evaluating PRP for knee OA, but there remains a lack of consensus due to factors such as study heterogeneity, differing PRP formulations, and study bias.^2,7,24^

In this study, we applied the FI and FQ to the literature on the use of PRP injections for the treatment of knee OA. The purpose of this study was to evaluate the fragility of randomized controlled trials (RCTs) evaluating the efficacy of PRP for knee OA with utilization of FI and FQ statistical analysis. We hypothesized that the overall FI and FQ would demonstrate significant statistical fragility with no difference appreciated between leukocyte-rich (LR) and leukocyte-poor (LP) formulations of PRP.

## Methods

This systematic review was conducted according to PRISMA guidelines. A comprehensive literature search of the PubMed database from 2000 to 2020 was performed using the search terms (*platelet rich plasma* OR *platelet rich products OR orthobiologics*) AND (*knee pain OR knee osteoarthritis*). Peer-reviewed RCTs pertaining to the use of PRP in knee OA in select journals with high impact factors were included for analysis (Table 1). The literature search was conducted independently by 2 reviewers (J.P.C and M.V.). Duplicate records were removed. Disagreements were resolved by referral to a third reviewer (D.W.).

**Table 1:** The 2019 Impact Factor of the Included Journals

Journal	Impact Factor
*American Journal of Sports Medicine*	5.810
*International Journal of Molecular Sciences*	4.556
*Arthroscopy*	4.325
*Journal of Orthopaedics and Traumatology*	2.767
*Clinical Rehabilitation*	2.599
*World Journal of Orthopedics*	0.798

**Figure 1.**
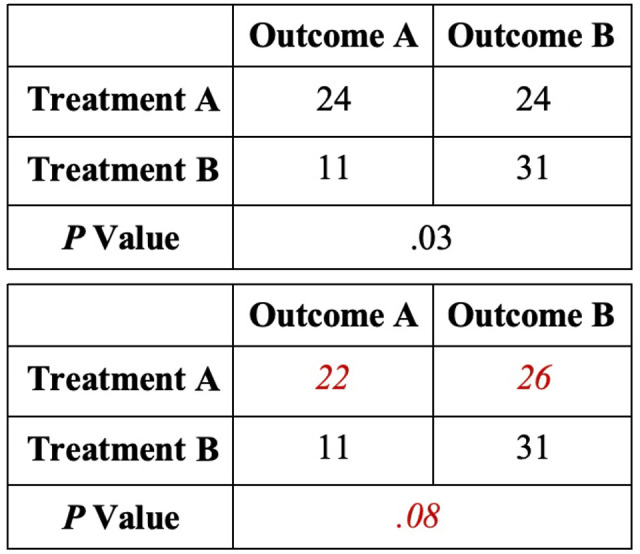
Example of a study with an outcome initially reported as statistically significant (top 2 × 2 contingency table). The number of outcome events required to change *P* to nonsignificant status (ie, the fragility index) was 2 (bottom 2 × 2 contingency table).

Included were RCTs that reported dichotomous comparative data with associated *P* value analysis. The type of outcome measure (primary, secondary, not specified) was documented. The particular formulation of PRP utilized was recorded as LR-PRP, LP-PRP, or not specified. Loss to follow-up (LTF) data were evaluated for all studies. Fragility analysis was performed by manipulating the reported outcome events in a 2 × 2 contingency table until a reversal of significance occurred, with statistical significance defined as *P* < .05 (Figure 1). For example, if a particular outcome was initially reported as statistically significant, the number of outcome events required to raise *P* to ≥.05 was determined. Conversely, if the outcome was initially reported as nonsignificant, the number of outcome events required to decrease *P* to <.05 was determined. The corresponding number indicates the number needed to reverse a particular outcome event and was recorded as the FI for that event.

All overturned outcome events were calculated in this manner, with the median value representing the FI for the entire study population. The associated FQ was determined for each outcome event by dividing the FI by the sample size. In addition, the total FQ for all outcome events was determined. The reported *P* value was recorded for each outcome event and verified for accuracy using the two-tailed Fisher exact test. Interquartile ranges (IQRs) were calculated to provide a more comprehensive understanding and interpretation of the reported variability and dispersion as the difference between the 25th and 75th percentiles.

## Results

**Figure 2.**
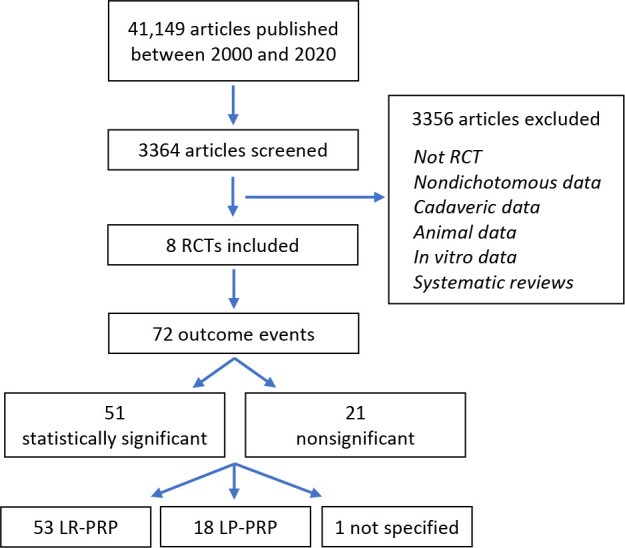
PRISMA study identification flowchart. LP, leukocyte poor; LR, leukocyte rich; PRP, platelet-rich plasma.

Of 41,149 initial studies, 3364 studies were screened, with 8 RCTs^4,16–19,22,23,25^ ultimately meeting both inclusion and exclusion criteria and thus included for analysis (Figure 2). There were 72 total outcome events, with 51 (70.8%) initially reported as statistically significant (*P* < .05) and 21 (29.2%) initially reported as not significant (*P* ≥ .05). Outcomes initially reported as significant were found to be vastly more stable than those initially reported as not significant. Of the 51 outcomes initially reported as statistically significant, the median number of events required to reverse significance (FI) was 12 (IQR 5-18) (Table 2). The associated FQ for statistically significant outcomes was 0.164 (IQR 0.078-0.273). Of the 21 outcomes initially reported as not statistically significant, the median number of events required to reverse significance (FI) was only 5 (IQR 1.5-7) with a mean *P* value of .51. The associated FQ for initially nonsignificant outcomes was 0.093 (IQR 0.022-0.148). Primary outcomes (n = 27) were slightly more fragile than secondary outcomes (n = 45) with an FI of 8 (IQR 5-14) and 9 (IQR 4-16.5), respectively. The associated FQ also supported slightly increased fragility with primary versus secondary outcomes with values of 0.111 and 0.164, respectively.

**Table 2:** Fragility Data Based on Trial and Outcome Characteristics

Characteristic	Events	Fragility Index (IQR)	Fragility Quotient (IQR)
All Trials	72	8.5 (4.5-14.5)	0.14 (0.066-0.218)
Outcome			
Primary	27	8 (5-14)	0.111 (0.056-0.156)
Secondary	45	9 (4-16.5)	0.164 (0.071-0.282)
Reported *P* value			
<.05	51	12 (5-18)	0.164 (0.078-0.273)
≥.05	21	5 (1.5-7)	0.093 (0.022-0.148)
PRP formulation			
LR-PRP	53	12 (6.5-17.5)	0.164 (0.090-0.264)
LP-PRP	18	3.5 (2-6)	0.061 (0.031-0.125)
Not specified	1	6	0.111

*^a^* Abbreviations: IQR, interquartile range; LP, leukocyte poor; LR, leukocyte rich; PRP, platelet-rich plasma.

Of the 8 RCTs analyzed, 4 (50%) represented data from LR-PRP formulations^16,22,23,25^ and 3 (37.5%) from LP-PRP formulations,^4,18,19^ with 1 study (12.5%) that did not specify leukocyte characteristics of the PRP used.^17^ The platelet concentration was reported in 2 (25%) of the included trials.^4,19^ Further fragility analysis of RCTs utilizing LR-PRP was found to be more stable than those utilizing LP-PRP. In evaluation of 53 LR-PRP and 18 LP-PRP outcome events, the FI was determined to be 12 (IQR 6.5-17.5) and 3.5 (IQR 2-6), respectively. Similarly, the FQ was 0.164 for LR-PRP and 0.061 for LP-PRP.

The overall FI, incorporating 72 outcome events from all 8 RCTs, was 8.5 (IQR 4.5-14.5). Accounting for sample size, the overall FQ was 0.14 (IQR 0.066-0.218), suggesting the reversal of 14% of outcome events is required to alter trial significance. All 8 RCTs reported loss to follow-up data.

## Discussion

Our analysis of all outcome events after PRP in the treatment of knee OA suggests a lack of statistical stability in the included RCTs, with an FI of 8.5 and an associated FQ of 0.14, suggesting the reversal of only 14% of events required to alter study significance and, thus, the conclusions found within the studies. When comparing LR-PRP and LP-PRP formulations, we found that the reported LR-PRP data were substantially more stable than the LP-PRP data.

The fragility findings of this review may be of particular concern, as outcomes of RCTs represent the best available data and are frequently utilized to inform clinical practice guidelines. Our findings provide further evidence in support of recent fragility analyses revealing statistical fragility in the greater orthopaedic literature.^5,10,20,21^ Furthermore, 1 of the 8 RCTs reported an LTF greater than the overall FI.^18^ If all the missing data were to skew in the same direction, it may be possible in this instance to realize a reversal of statistical significance by merely maintaining study follow-up. As such, it is important for academic and clinical professionals to appreciate the limitations of *P* value analysis by clearly distinguishing between statistical significance and clinical applicability. Therefore, we support previous recommendations for the presentation of the *P* value in conjunction with FI and FQ analysis to enhance understanding.

In evaluation of trial results initially reported as significant, we found they were considerably more stable than those reported as nonsignificant.^20,21^ The FI and FQ of the 51 outcomes reported as significant were 12 and 0.164, respectively. Comparatively, the FI of the 21 nonsignificant outcomes was only 5 with an associated FQ of 0.093. The latter represents the greater ease of reversal of potential false negative findings as compared to false positives. However, when we analyzed the trials individually, we found that the number of outcomes associated with significant and nonsignificant findings may explain the discrepancy.^20,21^ We found no consistent trend for the initial reported significance; rather, we found trials with more reported outcome events were generally more stable. In all included studies, increased statistical robustness via the FI correlated with cohort size. However, when normalizing with respect to sample size, the FQ for significant outcomes was 0.164 and provides further support of increased stability as compared with nonsignificant outcomes with an FQ of 0.093.

Statistical fragility is not a novel concept with growing research supporting the necessity of complementary statistics in the medical literature. Checketts et al evaluated 72 orthopaedic clinical trials cited as providing strong evidence in the American Academy of Orthopaedic Surgeons Clinical Practice Guidelines.^5^ They identified an FI of only 2 with an associated FQ of 0.022. The authors additionally noted an FI of 0 in 16 of the included trials as they demonstrated a reversal of statistical significance by calculating *P* values via the Fisher exact test. This highlighted alarming statistical fragility with 22% of all trials representing significant vulnerability to reversal with the simple use of an alternative statistical test for *P* value analysis.^5^ This further underscores the importance of reporting complementary statistics accompanying *P* values in order to provide a more complete picture of that statistical integrity of comparative trials.

Our findings regarding statistical fragility in RCTs on PRP for knee OA indicate relatively higher stability compared with findings in other orthopaedic subspecialties. In an analysis of 132 outcome events in 40 eligible RCTs pertaining to spine surgery interventions, Evaniew et al reported an FI of 2 with 65% of trials demonstrating an FI less than or equal to the reported LTF.^8^ Similarly, an analysis of orthopaedic oncology literature conducted by Forrester et al identified a median FI of 2 which was less than number LTF in 60% of outcomes.^10^ In addition, Khan et al analyzed the results from 48 RCTs from sports medicine and arthroscopic surgery literature and identified an FI of 2 (IQR 1-2.8).^14^ Furthermore, Khormaee et al performed a systematic evaluation of pediatric orthopaedic literature published over a 10-year period between 2006 and 2016.^15^ They analyzed 116 outcome events from 17 RCTs and reported an FI of 3.^15^ These findings further emphasize the advantage of FI and FQ metrics as easily identifiable statistical complements to the *P* value, providing a more complete understanding of trial stability. This is of particular importance in the treatment of knee OA as there remains conflicting evidence and a lack of consensus regarding the clinical efficacy of PRP.

### Limitations

Our study is not without limitations. We carefully evaluated RCTs examining the use of PRP for knee OA, but our findings may not be utilized to recommend for or against treatment. Furthermore, the included RCTs utilized differing PRP formulations with variable comparisons to corticosteroid or hyaluronic acid injections, so we therefore cannot suggest specific management recommendations. Rather, our fragility analysis provides an assessment of the statistical robustness of the highest level of evidence evaluating the management of knee OA with PRP formulations across all relevant RCTs reported in the literature. Given the lack of fragility analyses in the evaluation of PRP for knee OA, as well as across the greater orthopaedic literature, specific fragility thresholds have yet to be determined and require further study for future guidance on trial robustness and clinical decision-making.

## Conclusion

Comprehensive fragility analysis suggests the published literature evaluating the efficacy of PRP for knee OA may lack statistical stability. We therefore recommend the reporting of both an FI and FQ in addition to *P* value analysis to provide a clear and thorough understanding of the statistical integrity of studies reporting on PRP use for knee OA.
